# Macular Degeneration Drug Prescribing Patterns After Step Therapy Introduction in Medicare Advantage

**DOI:** 10.1001/jamahealthforum.2024.2446

**Published:** 2024-08-09

**Authors:** Angela Liu, Kelly E. Anderson, Joseph Levy, Thomas V. Johnson, Daniel Polsky, Gerard Anderson

**Affiliations:** 1 Department of Health Policy and Management, Johns Hopkins Bloomberg School of Public Health, Baltimore, Maryland; 2 Department of Clinical Pharmacy, University of Colorado Skaggs School of Pharmacy and Pharmaceutical Sciences, Aurora; 3 Wilmer Eye Institute, Johns Hopkins School of Medicine, Baltimore, Maryland; 4 Johns Hopkins Carey School of Business, Baltimore, Maryland

## Abstract

**Importance:**

In Medicare Advantage (MA), step therapy for physician-administered drugs is an approach to lowering drug spending. The impact of step therapy in MA on prescribing behavior and the magnitude of any changes has not been analyzed.

**Objective:**

To evaluate the impact of step therapy on macular degeneration drug prescribing patterns for 3 large MA insurers.

**Design, Setting, and Participants:**

This was a retrospective encounter-based analysis using 20% nationally representative MA outpatient and carrier encounter records for 2017 to 2019. Participants were MA beneficiaries who were 65 years or older and had received a macular degeneration drug administration. Macular degeneration drug administrations for beneficiaries of MA Aetna, Humana, and UnitedHealthcare (UHC) insurers were assessed. Humana implemented macular degeneration step therapy in 2019, setting bevacizumab as the plan-preferred drug, and aflibercept and ranibizumab as the plan-nonpreferred drugs. Aetna and UHC, which did not implement macular degeneration step therapy, served as the control group. Data analyses were performed from May 2024 to December 2024.

**Exposures:**

A macular degeneration drug administration subject to a step therapy policy.

**Main Outcome and Measures:**

A binary indicator of whether the drug administered was bevacizumab. Linear probability models and a difference-in-differences framework were used to quantify changes in prescribing patterns before and after the introduction of step therapy for MA insurers that did and did not implement step therapy. To empirically measure the impact of step therapy, the first administration of a treatment episode was assessed, followed by switching patterns.

**Results:**

A total of 18 331 MA beneficiaries, 21 683 treatment episodes, and 171 985 drug administrations were included across the control and treatment groups. The difference-in-differences regressions found a 7.8% (95% CI, 4.9%-10.7%; *P* < .001) greater probability of being prescribed bevacizumab for the first administration due to step therapy. The predicted probabilities of preferred-drug administration in the treatment group increased from 0.61 to 0.70 between the periods before and after step therapy implementation for the first administration. Step therapy was not significantly associated with an increased rate of medication switching (hazard ratio, 0.86; 95% CI, 0.71-1.06; *P* = .15).

**Conclusions and Relevance:**

The findings of this retrospective encounter-based analysis indicate that step therapy is associated with a greater probability of prescribing the plan-preferred drug for the first administration. The analysis failed to find a statistically significant greater rate of medication switching within a treatment episode. Step therapy changed macular degeneration prescribing patterns, but step therapy alone did not transition all administrations to the plan-preferred drug.

## Key Points

**Question:**

Does Medicare Advantage step therapy change prescribing patterns for macular degeneration physician-administered drugs?

**Findings:**

This retrospective encounter-based analysis including data from 171 985 macular degeneration drug administrations among a 20% nationally representative Medicare Advantage encounter sample revealed that from 2017 to 2019, step therapy was associated with a 7.8% greater probability of prescribing the plan-preferred drug for the first administration of a treatment episode. The predicted probability of being prescribed the plan-preferred drug for the treatment group increased from 0.61 before to 0.70 after the introduction of step therapy.

**Meaning:**

These findings suggest that step therapy for macular degeneration drugs slightly increases the prescribing of insurer-preferred products.

## Teaser

This retrospective encounter-based analysis evaluates the impact of step therapy on macular degeneration drug prescribing patterns among 3 large Medicare Advantage insurers.

## Introduction

The use of prior authorization as a strategy to manage health care utilization and limit health spending has been increasing.^1,2^ A type of prior authorization policy commonly used in the context of drug prescribing is step therapy. It requires beneficiaries to start with an insurer-designated plan-preferred drug therapy and progress to a plan-nonpreferred drug only if the plan-preferred option produces either a minimal clinical response, drug intolerance, or an adverse event.^3^ Step therapy has the potential to change drug prescribing patterns and to reduce drug spending, especially when several drug options of comparable clinical effectiveness are available but at substantially different cost. Although it intends to encourage high-value care, step therapy has generated concerns regarding health care access because patients may not always be able to obtain the drug that is most appropriate, promptly or at all.^4^ Additionally, step therapy has been criticized for (1) potentially undermining physician agency and harming the physician-patient relationship^5^; (2) allowing insurers to select the drug classes and drugs subject to step therapy without knowing the clinical circumstances of the individual patient (ie, insurers may focus on limiting the use of costly drugs, regardless of their clinical value, systemic adverse effects, ease and/or convenience of administration, or the possibility of drug-drug interactions)^6,7,8^; and (3) negatively impacting beneficiary health through reduced medication initiation or increased treatment switching.^9,10^ In response to these potential harms, multiple states have enacted patient protection policies to regulate step therapy for insurance products overseen at the state level.^11^

Step therapy for physician-administered drugs is not broadly permitted in Medicare and cannot be used in Traditional Medicare. However, federal rulemaking allowed for step therapy to be implemented by MA insurers starting on January 1, 2019, specifically on physician-administered drugs paid for through the Part B benefit.^3^ The US government reimburses MA insurers a per-member per-month capitated payment, and thus MA insurers are given financial incentives to limit health care spending, including on physician-administered drugs. Additionally, MA insurers can design physician networks^12,13,14^ that afford greater insurer control over clinicians, creating an environment in which step therapy has a higher chance of being followed by prescribers.

To our knowledge, this article is the first to examine whether implementation of step therapy by an MA insurer leads to associated behavior changes in the prescribing choices of physicians. Specifically, this study examines prescribing changes for drugs used to treat age-related macular degeneration. This is a best-case scenario for the cost reducing intention of step therapy due to the demonstrated clinical noninferiority and large cost differential between drugs. The 2 most prevalently prescribed macular degeneration antivascular endothelial growth factor drugs—aflibercept and ranibizumab—are among the highest spending by Traditional Medicare on physician-administered drugs.^15,16,17^ Evidence supports comparative clinical noninferiority among these 2 drugs and a third drug (bevacizumab)—available at approximately one-tenth of the cost—although bevacizumab is considered off-label for the treatment of age-related macular degeneration.^18,19,20^ Lastly, age-related macular degeneration mainly impacts older adults. That is, among those 50 years and older, macular degeneration prevalence is estimated to be as high as 19.5% for early-stage disease^21,22^ and is among the leading causes of irreversible blindness for this population.

Of large MA insurers, Humana implemented step therapy for macular degeneration drugs, designating bevacizumab as the plan-preferred drug, and aflibercept and ranibizumab as the plan-nonpreferred drugs. Aetna and UnitedHealthcare (UHC) did not implement step therapy for macular degeneration drugs. Therefore, this article evaluates physician prescribing patterns for Humana beneficiaries before and after the implementation of step therapy compared with prescribing patterns for beneficiaries insured by Aetna and UHC. First, this analysis focuses on the drug prescribed for the first administration of a beneficiary’s new treatment episode for macular degeneration. Second, this work examines drug switching over the course of a beneficiary’s treatment episode.

## Methods

This study was reviewed and approved by the Johns Hopkins Bloomberg School of Public Health Institutional Review Board (No. 11318). Informed consent was waived because this was a secondary analysis of an existing dataset. The study followed the Strengthening the Reporting of Observational Studies in Epidemiology (STROBE) reporting guidelines.^23^

### Data Collection

This retrospective encounter records-based analysis used a 20% nationally representative random sample of MA Part B outpatient and carrier encounter data for 2017 to 2019. These data allowed identification of Part B drug encounter records and associated diagnosis codes, as well as beneficiary, physician, hospital, and plan identifiers. MA plan enrollment details were drawn from the 2017 to 2019 Master Beneficiary Summary File. To identify plan type and MA insurer, publicly available “monthly enrollment by contract” data published by the US Centers for Medicare & Medicaid Services was used. Because these data are published monthly, the June data for each of the respective years was used—contract identification number, plan type, and plan name remain the same for the duration of the year. Using the column *plan type*, plans were identified as a preferred provider organization (PPO, identified as either local PPO or regional PPO) or health maintenance organization (HMO, identified as either HMO or HMO Point of Service). To identify the specific insurer, a combination of the organization’s name, marketing name, and parent organization were used. Insurer-level macular degeneration step therapy policies for 2019 were manually collected from publicly available documents published by the individual insurers.

### Study Sample

Beneficiaries were included who were 65 years and older, residents of the 50 US states or Washington, DC, and continuously enrolled in a Humana, Aetna, or UHC MA HMO or PPO plan throughout a given year or until death. To identify beneficiaries who were administered drugs for treatment of macular degeneration, we used the following codes from the Healthcare Common Procedure Coding System included in the 2017 to 2019 Medicare Table of Drugs: J1078 for aflibercept; J2778 for ranibizumab; and C9256, J7999, and J9035 for bevacizumab. Bevacizumab has multiple indications and is used to treat macular degeneration off-label; therefore, encounter records were restricted to those containing a recorded diagnosis code starting with H35.32, indicating “exudative age-related macular degeneration,” in any listed diagnoses, regardless of order (eTable 1 in Supplement 1).

### Treatment Episodes

A treatment episode was defined as at least 1 administration of a macular degeneration drug and included all administrations after the first when the subsequent administration(s) occurred within 150 days of the previous administration. Thus, new treatment episodes were defined as a beneficiary starting a macular degeneration drug treatment after a 150-day washout period. The number of beneficiaries and treatment episodes do not have a 1 to 1 mapping because beneficiaries can start a treatment episode, have a 151-day period without an administration, and/or start a second distinct treatment episode.

Although step therapy is intended to guide an entire treatment episode, after the first administration, physicians may switch drug products under certain circumstances. Thus, the association of step therapy with prescribing patterns was evaluated for both the first administration of a treatment episode, as well as for subsequent administrations by examining drug switching patterns.

### Empirical Strategy

#### Independent and Outcome Variables

The main explanatory variable in the difference-in-differences (DiD) design was the implementation of Part B step therapy for macular degeneration drugs. Humana implemented step therapy for macular degeneration treatments in 2019, and designated aflibercept and ranibizumab as the plan-nonpreferred drugs and bevacizumab as the plan-preferred drug (eTable 2 in Supplement 1). Aetna and UHC implemented step therapy for other treatments but did not require step therapy for macular degeneration drugs during the study period. The main outcome variable was a binary indication of whether the drug administered was bevacizumab.

#### First Administration

To assess the impact of step therapy on the prescribing choice for a new treatment episode, the sample was restricted to the first administration of a treatment episode. An event study was used to assess prescribing patterns at the quarterly level. Linear probability models and a DiD framework were used to quantify the effect of step therapy intervention on the drug prescribing. Parallel trends between the control and treatment group were visually inspected using a traditional parallel trend plot, and were then further examined by interacting the time effects with the group indicator. To account for the role of the facility (hospital or physician office) in drug prescribing choices, the model was run with an additional fixed effect on facility, using the organization’s National Provider Identification number or tax identification number.

#### Medication Switching

To evaluate medication switching, all administrations of a treatment episode were included. Time-to-event Kaplan-Meier survival curves were used to visually inspect switching between the treatment and control groups for treatment episodes that originated in 2017 and 2018 compared to 2019. Within the Kaplan-Meier survival function framework, failure was defined as a drug switching event. Because the maximum number of days in a look-forward period were not equal for treatment episodes originating in 2017 (1095 days or 3 years) and treatment episodes originating in 2019 (365 days or 1 year), treatment episodes were subset to a 365-day look-forward period to ensure consistency between the 2 groups.

Leveraging a DiD framework, a cox hazard ratio regression was used to quantify the effect of step therapy on medication switching within a beneficiary’s treatment episode. The functional form of the model is provided in the eAppendix in Supplement 1 along with tests for preperiod parallel trends and proportional hazards. Given that Humana’s step therapy policy required bevacizumab as the plan-preferred drug, subsetting to treatment episodes that start with bevacizumab examined the type of medication switching of greatest concern and was our primary specification. This examines whether a higher share of Humana administrations subsequently switched away from bevacizumab because it is not the drug either they or their physician preferred, absent step therapy requirements. Using methods developed by Jung et al,^24^ we calculated the estimated savings due to step therapy.

#### Sensitivity Checks

First, the association of step therapy with drug prescribing was tested using 2 washout period definitions: 90-day and 365-day. Second, because the literature^24^ suggests incomplete reporting for MA encounter data, data were restricted to plans with high levels of data reporting as a sensitivity check. Third, we adjusted for beneficiary-, plan-, and geography-level covariates. Beneficiary variables included age, sex, race and ethnicity (per the Research Triangle Institute race code), and whether the beneficiary was eligible for both Medicare and Medicaid (dual eligible); plan variables included HMO or PPO and whether the contract had a special needs plan; geography variables included census region and inclusion in a core-based statistical area (inside or outside) (eTable 3 in Supplement 1). Finally, as a falsification test, the years of the study were changed to include 2016 to 2018. Given that step therapy was first allowed on January 1, 2019, the new time period did not include any years during which step therapy was allowed.

Statistical tests were 2-tailed and *P* values < .05 were considered statistically significant. Data analyses were performed from May 2024 to December 2024 using Stata, release 16 (StataCorp LLC).

## Results

### Treatment Episode Sample Characteristics

The study sample included 18 311 unique beneficiaries, 21 683 treatment episodes, and 171 985 drug administrations for aflibercept, ranibizumab, or bevacizumab during the study period. Table 1 provides a count of beneficiaries, treatment episodes, and administrations by groups; 8.8% of beneficiaries had multiple treatment episodes.

**Table 1.** Sample Size by Control and Treatment Groups and Before and After Step Therapy Introduction

Study sample	No.				
	Control group		Treatment group		Total^a^
	Before	After	Before	After	
Unique beneficiaries taking macular degeneration drugs	8782	5392	3333	2436	18 331
Treatment episodes	9885	5572	3714	2512	21 683
Administrations of macular degeneration drugs	109 392	26 652	26 665	9276	171 985
Bevacizumab prescribed for first administration of treatment episode, %	63.5	63.7	61.2	69.2	NA

Abbreviation: NA, not applicable.

^a^ Sum of the before and after periods and control and treatment groups. Row 1 shows the number of unique beneficiaries for each group and the total of unique beneficiaries. Given that 8.8% of the beneficiaries had more than 1 treatment episode, to ensure that the total number of beneficiaries was equal to the sum of the before and after periods and that control and treatment groups, unique beneficiaries is specified. Because treatment episodes and administrations cannot be repeated, the total is the straightforward summation for treatment episodes and administrations.

### Step Therapy and First Administration

**Figure 1.**
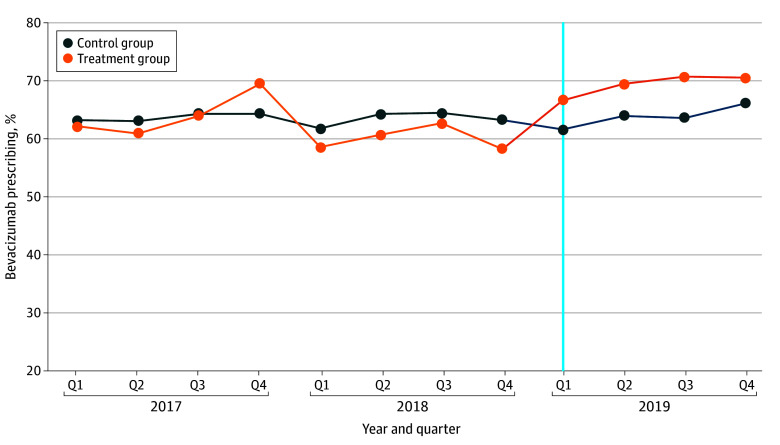
Unadjusted Percentage of Bevacizumab Administrations, 2017 to 2019 X-axis refers to the period from 2017 quarter (Q) 1 through 2019 quarter 4. The vertical line indicates the quarter for which step therapy was implemented for the treatment group, 2019 quarter 1. The labels indicate the percentage of bevacizumab drug administered for that quarter, defined as the number of bevacizumab administrations divided by the total number of macular degeneration drug administrations. The control group includes Aetna and UnitedHealthcare drug administrations, and the treatment group includes Humana drug administrations.

For the first administration of a treatment episode, the control group was prescribed bevacizumab 63.5% and 63.7% of the time, before and after implementation of macular degeneration step therapy (to which they were not subjected), respectively. Within the treatment group, bevacizumab was prescribed 61.2% and 69.2% of the time in the before and after periods, respectively, demonstrating an 8.0−percentage point increase in bevacizumab prescribing after step therapy implementation (Table 1 and Figure 1).

**Figure 2.**
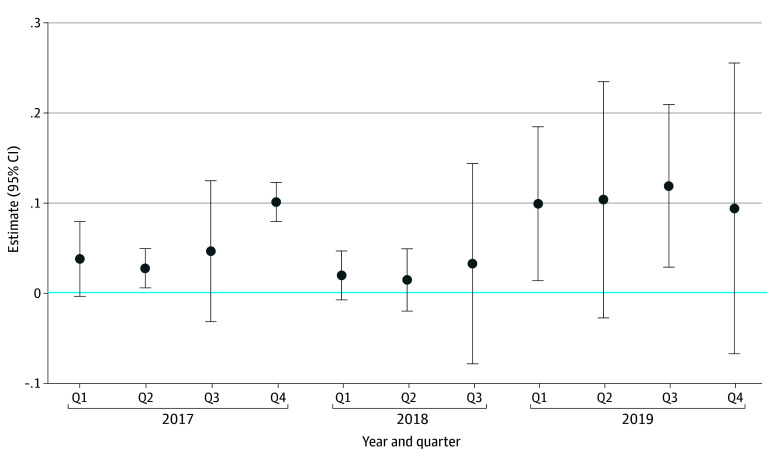
Event Study of Step Therapy on Bevacizumab Prescribing at the Annual Quarter Level Excludes 2018 quarter (Q) 4 because that time period directly preceded the implementation of step therapy. In 2017 and 2018, prescribing of bevacizumab was relatively constant, with the exception of 2017 quarter 4. After implementation of step therapy, the prescribing of bevacizumab increased.

Figure 2 shows the results of the event study at the quarter level.^25,26^ The primary DiD analysis showed there was a 7.8% (95% CI, 4.9%-10.7%; *P* < .001) greater probability of being prescribed bevacizumab during the before compared with the after period between the treatment and control groups regarding the first administration. This translates into an increased predicted probability of being prescribed bevacizumab, from 0.61 to 0.70, for the treatment group. We observed parallel trends in the before period (Figure 1). Additionally, when interacting time effects with the group indicator, we found that the coefficients in the before period were not statistically significant (eTable 4 in Supplement 1). Thus, we failed to reject the null hypothesis that the parallel trends assumption was violated.

**Figure 3.**
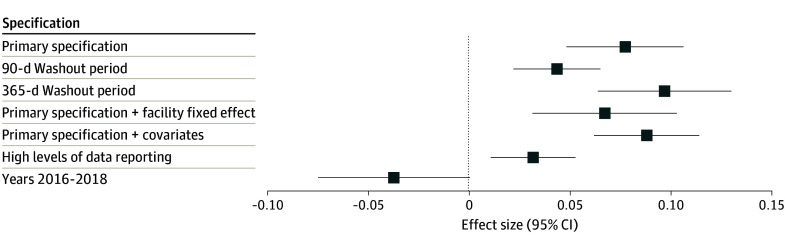
Coefficient Plot for Primary Specification and Sensitivity Checks Coefficient on the interaction term for the primary specification, which was an unadjusted model with treatment episodes specified with a 150-day washout period. Results of the sensitivity checks, including treatment episodes specified with a 90-day washout period, 365-day washout period, the primary specification with facility fixed effects included, the primary specification with covariates included (Supplement 1 provides covariate information), and when subset only to data with high levels of data reporting. The last specification included only years 2016 through 2018, when step therapy was not applied.

Figure 3 shows that findings were similar when examining a 90-day and 365-day washout period, when data were restricted to plans with a high level of data reporting, and when adjusted for beneficiary, plan, and geography-level covariates. Beneficiary and plan covariates were balanced across the groups and time periods. The treatment group (Humana) operated mainly in the US South region (66% of beneficiaries) compared to the control group, which was more balanced across census regions (eTables 3 and 5 in Supplement 1). Of note, when adjusting for beneficiary characteristics, the Humana dual-eligible population and not dual-eligible population, 82.2% and 67.4% received the plan-preferred drug for the first administration after the implementation of step therapy, respectively. eFigure 6 in Supplement 1 shows that physicians prescribed bevacizumab at a higher percentage for beneficiaries enrolled in Humana HMOs compared to Humana PPOs. Adding on a facility fixed effect, the interaction term dropped to 6.6% (95% CI, 4.2%-9.0%; *P* < .001), suggesting that 1.2 percentage points (or 15.4%) of the finding was driven by the particular facility where the drug was administered. The falsification test resulted in a nonstatistically significant interaction term (−3.7; 95% CI, −7.4 to 0; *P* = .06), lending credibility to the primary analysis that step therapy was associated with prescribing changes in the time period during which it was implemented.

### Step Therapy and Treatment Switching

Within Humana, the insurer that implemented step therapy, there were a total of 6226 treatment episodes: 3714 treatment episodes were initiated in 2017 or 2018 and were not subject to step therapy, and 2512 treatment episodes were initiated in 2019 and were subject to step therapy (eTable 6 in Supplement 1). Using equality of survivor log-rank test, the rate of failure differed between the treatment episodes subject to step therapy and not subject to step therapy, with switching occurring at a higher rate for the treatment episodes that began in 2019 (eFigure 2 in Supplement 1). The implication of this finding is that within Humana, there was a greater rate of switching for treatment episodes that started in 2019 compared to treatment episodes that started before 2019. However, the cox hazard ratio (HR) regression, which accounts for a control group, found that the interaction term between time and treatment group was not statistically significant (HR, 0.86; 95% CI, 0.71-1.06; *P* = .15), meaning that the rate of switching did not differ due to the implementation of step therapy, but rather both the treatment and control groups experienced a higher rate of switching in 2019 (Table 2). We tested parallel trends in the before period and the assumption of proportional hazards (eFigures 3 and 4 in Supplement 1). Taken together, these findings reveal an estimated $1.9 million in savings for Humana in 2019 or 11.8% of the insurer’s spending on macular degeneration drugs (eTable 7 in Supplement 1).

**Table 2.** Cox Hazard Ratio (HR) Regression Results for 3 Models, by First Drug Used in Treatment Episode

Variable	Model, by first drug in treatment episode		
	(1) Bevacizumab^a^	(2) Aflibercept	(3) Ranibizumab
Interaction term	0.86 (0.71-1.06)	2.64 (1.41-4.96)	1.42 (0.64-3.16)
*P* value	.15	.002^b^	.39
Post	1.36 (1.22-1.52)	0.59 (0.40-0.88)	1.08 (0.72-1.60)
*P* value	.001^c^	.01^d^	.72
Treatment	1.06 (0.94-1.18)	0.93 (0.67-1.28)	1.27 (0.87-1.85)
*P* value	.36	.64	.21
Administrations, No.	38 511	11 047	7258

^a^ The primary specification was model 1, which was subset to treatment episodes that started with bevacizumab. The HR for the interaction term is not statistically significant. To interpret the HR: after the introduction of step therapy, treatment episodes that started with bevacizumab were 13.6% less likely to switch away from bevacizumab within a treatment episode; however, this finding was not statistically significant.

^b^ *P* < .01.

^c^ *P* < .001.

^d^ *P* < .05.

## Discussion

The study findings indicate that macular degeneration Part B step therapy is associated with a 7.8% greater probability in prescribing bevacizumab for the first administration of a treatment episode among Humana MA beneficiaries, and that step therapy does not impact switching patterns for later administrations. Theoretically, step therapy could result in 100% of first administrations being the plan-preferred drug. However, even after Humana implemented step therapy, 30% of all incident administrations were for the plan-nonpreferred drug, indicating that step therapy alone does not transition all prescribing patterns to the plan-preferred drug product. Greater adherence to step therapy could save more money for insurance companies, but a continued look at patient well-being would be necessary because it is not clear whether the other more diverse or marginalized groups of beneficiaries would face negative consequences.

Several reasons may contribute to why step therapy alone does not transition all prescribing patterns to the plan-preferred drug product. First, we examined the first year of step therapy administration, and details around the implementation of step therapy may influence its impact. Physicians may not know that the beneficiary was subject to step therapy. Streamlining step therapy policies into electronic systems may mitigate this issue. Additionally, a beneficiary may not know that the drug of choice is not covered by insurance at the point of administration; only after receiving it may they discover their financial responsibility. Alternatively, another beneficiary may be willing to pay for the higher cost of the preferred drug. Second, physicians and beneficiaries can appeal step therapy. A successful appeal would allow physicians to prescribe their drug of choice during the first administration and throughout the treatment episode. Learning how to navigate the appeals process creates an additional burden on physicians and beneficiaries and may accentuate disparities in outcomes related to the social determinants of health. Specific populations, such as beneficiaries who have higher levels of health literacy and can better advocate for themselves, may be better positioned to successfully navigate the appeals process.

The results of this analysis suggest that through step therapy, insurers are able to treat a share of beneficiaries with plan-preferred drugs for macular degeneration. The question then arises, why are some insurers not implementing and using macular degeneration step therapy? MA insurers may be considering other factors when choosing to engage with step therapy. Future research should focus on whether MA beneficiaries leave MA plans due to step therapy or whether physicians choose not to contract with MA plans due to their lack of agency under step therapy policies or concerns regarding treatment outcomes.

The clinical implications of this work, although outside the scope of these analyses, are important to consider. While studies have demonstrated the clinical noninferiority of bevacizumab to ranibizumab and aflibercept,^18,19,20^ these randomized clinical studies were limited in their ability to generalize to the broader population outside of their study samples and dosing protocols, and were not powered to assess long-term effects, ie, lifetime impacts. Although comparable effects on visual acuity have been reported, beneficiaries may experience differential complication rates between drug products, which may be compounded by varying dosing intervals associated with the drugs. Indeed, aflibercept dosed every 2 months after 3 initial monthly doses achieves similar functional and structural efficacy as monthly ranibizumab.^27^ Patients who receive a greater number of intravitreal injections are subject to an increasing cumulative risk of complications. New drugs (faricimab) and formulations (high-dose aflibercept) have been introduced since this dataset was generated and may facilitate even less frequent dosing.

Recently, bevacizumab biosimilars have become available on the market, including bevacizumab-tnjn (2023), bevacizumab-adcd (2022), and bevacizumab-maly (2022). To our knowledge, the bevacizumab biosimilars have not been subject to equally rigorous study in the context of macular degeneration^28^; therefore, future work should consider bevacizumab biosimilars as a potential opportunity for additional savings for macular degeneration treatment.

### Limitations

There are 3 study limitations of note. First, the findings cannot be generalized beyond the clinical scenario of macular degeneration or beyond the sample of MA insurers included in this analysis. Second, linear probability models can generate predicted probabilities outside a reasonable bound (0 and 1). In this work, coefficients generated from the linear probability model were carefully examined to ensure clinical relevance, and the model was chosen for its interpretability of results. Third, the administrative encounter data provide little ability to study patient-centered outcomes or explore qualitative experiences related to step therapy. While qualitative research is critical future work, this analysis provides empirical evidence responding to first-order questions regarding step therapy’s impact on drug selection.

## Conclusions

The findings of this retrospective analysis indicate that implementation of step therapy in MA was associated with changes in prescribing patterns for macular degeneration physician-administered drugs. Specifically, in the presence of step therapy, there was a greater probability of prescribing the plan-preferred drug. Future research should determine whether these findings generalize to other drug classes that are subject to step therapy, particularly those involving drugs that have not demonstrated clinical noninferiority.

## Notes

Supplement 1. eAppendix. Supplemental Methods and Results eTable 1. Macular Degeneration HCPCS codes and associated ICD-10 diagnosis codes eTable 2. Macular Degeneration step therapy policy, 2019 eTable 3. Examining multi-level covariates, first administration of treatment episode eTable 4. Testing Parallel Trends Pre-Period Assumption eTable 5. Adjusted difference-in-difference regression for the first administration of a treatment episode eFigure 1. Macular degeneration prescribing patterns for Humana MA contracts by HMO or PPO eTable 6. Treatment episode characteristics eFigure 2. Kaplan-Meier Survivor Curves eFigure 3. Parallel Trends Pre-Period Testing for Cox Hazard Regression Model eFigure 4. Log-log plot to assess proportional hazards assumption eTable 7. Cost-savings calculations

Supplement 2. Data Sharing Statement
