# Supplementary material for: Macular Degeneration Drug Prescribing Patterns After Step Therapy Introduction in Medicare Advantage
Source: JAMA Health Forum. 2024 Aug 9;5(8):e242446. doi: 10.1001/jamahealthforum.2024.2446 (PMC11316235; doi:10.1001/jamahealthforum.2024.2446)
Supplement: Supplement 1. — eAppendix. Supplemental Methods and Results eTable 1. Macular Degeneration HCPCS codes and associated ICD-10 diagnosis codes eTable 2. Macular Degeneration step therapy policy, 2019 eTable 3. Examining multi-level covariates, first administration of treatment episode eTable 4. Testing Parallel Trends Pre-Period Assumption eTable 5. Adjusted difference-in-difference regression for the first administration of a treatment episode eFigure 1. Macular degeneration prescribing patterns for Humana MA contracts by HMO or PPO eTable 6. Treatment episode characteristics eFigure 2. Kaplan-Meier Survivor Curves eFigure 3. Parallel Trends Pre-Period Testing for Cox Hazard Regression Model eFigure 4. Log-log plot to assess proportional hazards assumption eTable 7. Cost-savings calculations [file jamahealthforum-e242446-s001.pdf]

Supplemental Online Content

Liu A, Anderson KE, Levy J, Johnson TV, Polsky D, Anderson G. Macular Degeneration Drug Prescribing Patterns After Step Therapy Introduction in Medicare Advantage. *JAMA Health Forum.*2024;5(8):e242446. doi:10.1001/jamahealthforum.2024.2446

eAppendix. Supplemental Methods and Results

eTable 1. Macular Degeneration HCPCS codes and associated ICD-10 diagnosis codes

eTable 2. Macular Degeneration step therapy policy, 2019

eTable 3. Examining multi-level covariates, first administration of treatment episode

eTable 4. Testing Parallel Trends Pre-Period Assumption

eTable 5. Adjusted difference-in-difference regression for the first administration of a treatment episode

eFigure 1. Macular degeneration prescribing patterns for Humana MA contracts by HMO or PPO

eTable 6. Treatment episode characteristics

eFigure 2. Kaplan-Meier Survivor Curves

eFigure 3. Parallel Trends Pre-Period Testing for Cox Hazard Regression Model

eFigure 4. Log-log plot to assess proportional hazards assumption

eTable 7. Cost-savings calculations

This supplemental material has been provided by the authors to give readers additional information about their work.

© 2024 Liu et al. *JAMA Health Forum*.

eAppendix.

Supplemental Methods

The supplemental methods provide more details regarding the adjusted linear probability models and the time-to-event models.

Adjusted Linear Probability Models

For adjusted analyses, this study leveraged beneficiary-, plan-, and geography-level covariates. Beneficiary demographic information and MA plan enrollment details were drawn from the 2017-2019 Master Beneficiary Summary File (MBSF). Plan- and geography-level covariates were identified using the MA plan enrollment details drawn from the MBSF. Supplemental Exhibit 3 examines the covariate balance by pre- and post- period and by control and treatment groups.

Time-to-Event Analysis

The time-to-event analysis relies on the cox hazard regression model within the difference-in-difference framework. The functional form of the model is presented below:

$$\log(H(t)) = H_0(t) + \beta_1 Post_t + \beta_2 Treatment_i + \beta_3\,(Post_t\ x\ Treatment_i)$$

Where:

$H(t)$: expected hazard at time, *t;* $H_0(t)$: baseline hazard and represents the hazard when all predictors are equal to 0; $Treatment_i \in [0,1]$: administration, i, as either treatment or control MA insurer group; and $Post_t \in [0,1]$: quarter, *t,* as either pre (2017 and 2018 combined) or post (2019).

To test the parallel trends assumption, we generate hazard functions for the pre period for the control and treatment groups separately. We then graph the smoothed hazard estimates to examine trends in the pre-period (Supplemental Exhibit 9). To test for the assumption of proportional hazards, we visually assess the log-log plot for the control and treatment group (Supplemental Exhibit 10). Next, we perform a goodness-of-fit test by checking the correlation between the Schoenfeld residuals and time to see if there is a significant relationship.

Supplemental Results

Adjusted Linear Probability Models

Results from the robustness checks can be seen in the coefficient plot in Figure 3. While the primary specification defines a new treatment episode with a 150-day washout period, 90-day and 365-day washout periods were additionally tested. The a priori hypothesis is that the smaller the washout period, the smaller the effect size. A smaller washout period indicates a less conservative approach to defining new treatment periods, meaning that a smaller washout period will be less sensitive in correctly identifying new treatment episodes as new, rather than as a continuation of treatment. Since step therapy requires the plan-preferred drug to be administered for new administrations, the effect is hypothesized to be smaller. When using a 90-day washout period, the interaction term is 4.4 (95% CI: 2.2-6.5; p<0.001); when using a 365-day washout

© 2024 Liu et al. *JAMA Health Forum*.

period, the interaction term is 9.7 (95% CI: 6.4-13.0; p<0.001). Since the results of these checks are in the direction hypothesized, these checks lend strength to our finding in the primary specification.

Additional robustness checks incorporate facility fixed-effects, multi-level covariates, and finally limit the data to plans with high-levels of data reporting to account for potential data limitations with the MA encounter data. When adding a facility fixed effects, the interaction term is 6.4 (95% CI: 2.9-10.0; p<0.001); when adding in multi-level covariates, the interaction term is 8.4 (95% CI: 4.8-11.0; p<0.001); when restricting data to plans with high-levels of data reporting, the interaction term is 3.2 (95% CI: 1.1-5.3; p<0.001). Supplemental Exhibit 5 depicts the results of the fully adjusted regression.

Time-to-Event Analysis

Supplemental Exhibit 9 visually suggests that the pre-period parallel trends assumption is met. Supplement Exhibit 10 shows the log-log plot of the control and treatment groups after running the cox hazard regression. We visually see that there is not strong evidence of non-parallelism of the log-log curves. Further, we perform a goodness-of-fit test by checking the correlation between the Schoenfeld residuals and time to see if there is a significant relationship. We find a non-significant p-value (p=0.4217) which suggests that the proportional hazards assumption holds.

© 2024 Liu et al. *JAMA Health Forum*.

eTable 1. Macular Degeneration HCPCS codes and associated ICD-10 diagnosis codes

Drug	HCPCS codes	ICD-10 codes for macular degeneration
Bevacizumab	C9256, J7999, J9035	H35.32, H35.321, H35.3210, H35.3211, H35.3212, H35.3213, H35.322, H35.3220, H35.3221, H35.3222, H35.3223, H35.323, H35.3230, H35.3231, H35.3232, H35.3233, H35.329, H35.3290, H35.3291, H35.3292, H35.3293
Aflibercept	J0178	n/a
Ranibizumab	J2778	n/a

Note: This exhibit depicts the HCPCS codes used to identify the physician-administered drugs included in the sample. It further depicts the ICD-10 codes used to identify encounters in which bevacizumab was prescribed for macular degeneration.

HCPCS = Healthcare Common Procedure Coding System; ICD-10 = International Classification of Diseases-10

© 2024 Liu et al. *JAMA Health Forum*.

eTable 2. Macular Degeneration step therapy policy, 2019

Indication	Drug	Plan step therapy designation	Price/dose	Dosing schedule	Treatment Group	Control Group
Macular Degeneration	Bevacizumab	Plan-preferred	$72	1x/~4-6 weeks	Humana	Aetna, United
	Aflibercept	Plan-non-preferred	$932			
	Ranibizumab	Plan-non-preferred	$352			

Note: The "price/dose" column was populated using the publicly available Medicare Part B dashboard.

© 2024 Liu et al. *JAMA Health Forum*.

eTable 3. Examining multi-level covariates, first administration of treatment episode
This table shows the distribution of beneficiary-, plan-, and geography-level covariates between the control and treatment groups in the pre and post period. The distribution is balanced for beneficiary and plan characteristics. We observe that Humana mainly operates in the South, whereas the control group MA insurers (Aetna and United) are more balanced in their geography census region distribution.

	Pre-period		Post-period	
	Control	Treatment	Control	Treatment
Beneficiary				
Age of beneficiary	82.3 (SD 7.6)	81.2 (SD 7.5)	82.2 (SD 7.6)	81.1 (SD 7.6)
Sex of beneficiary				
Female	64%	61%	63%	62%
Male	36%	38%	37%	38%
Race of beneficiary				
American Indian	0.2%	0.2%	0.2%	0.45%
Asian/Pacific Islander	2.6%	1.4%	4.0%	1%
Black	2.0%	4.0%	3.0%	5%
Hispanic	6.6%	5.5%	7.0%	8%
Non-Hispanic White	87.5%	88.0%	85.0%	85%
Other	0.7%	0.4%	0.5%	0.58%
Unknown	0.4%	0.5%	0.5%	0.5%
Whether the beneficiary is dual-eligible	10.4%	11.2%	11.2%	12.1%
Plan				
HMO or PPO				
HMO	57.8%	55.4%	52.7%	50.9%
PPO	42.2%	44.6%	47.3%	49.1%
Whether contract has SNP	40.2%	55.2%	48.8%	50.9%
Geography				
CBSA category, inside or outside				
Inside	90.1%	83.8%	90.3%	83.9%
Outside	9.9%	16.2%	9.7%	16.1%
Census Region				
Midwest	19.2%	19.4%	18.4%	19.7%
Northeast	15.1%	2.7%	16.7%	2.6%
South	27.0%	68.8%	29.9%	66.0%
West	38.7%	9.1%	35.1%	11.7%

Note: The table examines multi-level covariates at the beneficiary, physician, plan, and geography level. HMO= Health Maintenance Organization, PPO=Preferred Provider Organization, SNP=Special Needs Plans, CBSA=Core Based Statistical Areas.

© 2024 Liu et al. *JAMA Health Forum*.

eTable 4. Testing Parallel Trends Pre-Period Assumption

	Coefficient	P-value
**Treatment**	-0.011	p=0.682
**Quarter**		
Quarter 2	-0.001	p=0.951
Quarter 3	0.011	p=0.567
Quarter 4	0.011	p=0.586
Quarter 5	-0.015	p=0.415
Quarter 6	0.011	p=0.568
Quarter 7	0.012	p=0.527
Quarter 8	0	p=0.966
**Treatment*Quarter**		
Treated*Quarter 2	-0.011	p=0.795
Treated*Quarter 3	0.008	p=0.856
Treated*Quarter 4	0.063	p=0.132
Treated*Quarter 5	-0.021	p=0.553
Treated*Quarter 6	-0.025	p=0.493
Treated*Quarter 7	0.007	p=0.858
Treated*Quarter 8	-0.039	p=0.296

Note: We create dummy variables for each of the quarters in the pre-period, and interact the dummy variables with the group variable. The resulting coefficients are not statistically significant, suggesting that there is no systematic difference in trends between the control and treatment groups in the pre-treatment period, which supports the parallel trends assumption.

© 2024 Liu et al. *JAMA Health Forum*.

eTable 5. Adjusted difference-in-difference regression for the first administration of a treatment episode

This exhibit provides the results of the adjusted linear probability model for beneficiary-, plan-, and geography-level coefficients. We find that after adjusting for covariates, the interaction coefficient is in the same direction and of similar magnitude toe the primary specification with no covariates. As expected, the census region has the largest coefficient, as the MA insurers in the control and treatment group are not balanced across census region. For example, there is a 24.6% greater probability of being prescribed bevacizumab in the South compared to the Midwest. This is as expected since Humana mainly operates in the South.

		Probability of plan-preferred drug administration (95% CI)	
Time		3.8***	(2.4-5.1)
Treatment		-4.7***	(-6.6 to -2.9)
Interaction Term		8.4***	(4.8-11.0)
Beneficiary			
Age		-0.0	(0-0)
Dual eligibility (reference: non-duals)		4.9***	(2.1-5.8)
Sex (reference: males)		0	(0-0)
Race of beneficiary (reference: Non-Hispanic White)			
	American Indian	3.0	(-9.4-15.4)
	Asian/Pacific Islander	4.6*	(0-8.4)
	Black	3.9*	(0-7.3)
	Hispanic	9.0***	(6.5-11.4)
	Other	6.7	(-1-14)
	Unknown	-8.1*	(-.2 to 0)
Plan			
Plan Type (reference: HMO)		-8.0***	(-9.3 to -6.6)
Whether contract has SNP (reference: no SNP)		2.8***	(1.2-4.3)
Geography			
CBSA category (reference: Outside CBSA)			
	Inside	-0.7	(-2.2-0)
	Unknown	2.3	(-10-5.6)
Census Region (reference: Midwest)			
	Northeast	3.9***	(2.1-5.8)
	West	10.8***	(9.4-12.2)
	South	24.6***	(21.8-26.4)

*p<0.05, **p<0.01, ***p<0.001

Note: HMO=Health Maintenance Organization; SNP=Special Need Plan; CBSA=Core-based Statistical Area

© 2024 Liu et al. *JAMA Health Forum*.

eFigure 1. Macular degeneration prescribing patterns for Humana MA contracts by HMO or PPO

In this exhibit, we graph the percentage of administrations for bevacizumab. We observe through this figure that HMOs prescribe bevacizumab at a higher rate than PPOs, suggesting that plan type plays a role in prescribing choice.

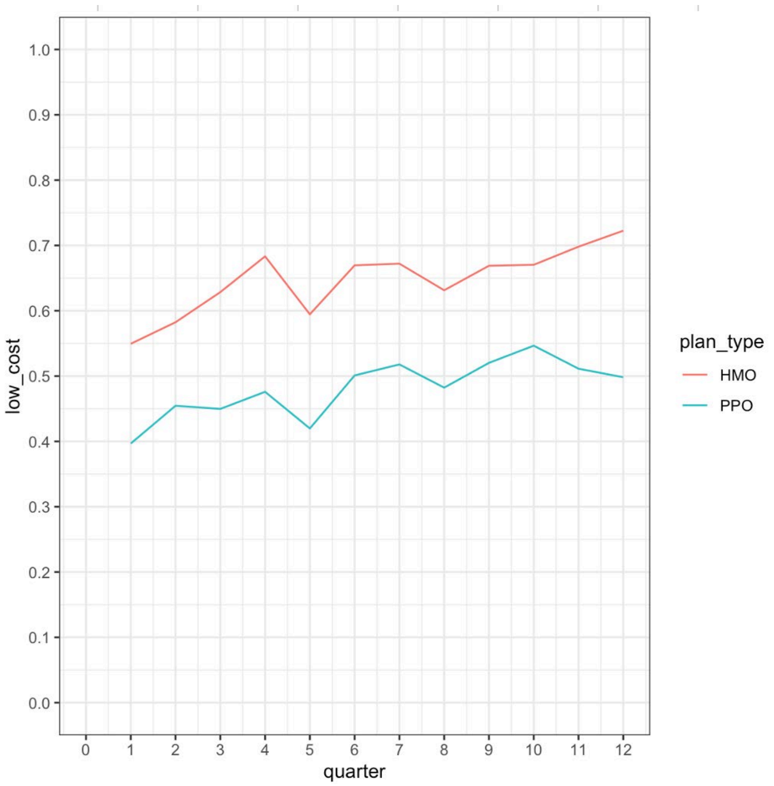

Note: The y-axis is showing the percentage of administrations for bevacizumab. The red line is for Humana health maintenance organizations and the blue line is for Humana preferred provider organizations.

HMO = Health Maintenance Organization; PPO = Preferred Provider Organization

© 2024 Liu et al. *JAMA Health Forum*.

eTable 6. Treatment episode characteristics

	Control		Treatment	
	Pre-period	Post-period	Pre-period	Post-period
Number of treatment episodes	9,885	5,572	3,714	2,512
Starting with…				
Bevacizumab	6,278 (63.5%)	3,551 (63.7%)	2,275 (61.2%)	1,739 (69.2%)
Aflibercept	1,880 (19.0%)	1,124 (20.2%)	592 (15.9%)	369 (14.7%)
Ranibizumab	1,904 (19.3%)	642 (11.5%)	322 (8.7%)	114 (4.5%)
Average # of administrations per treatment episode, Mean (SD)	5.3 (6.8)	2.8 (2.6)	4.0 (4.2)	2.3 (1.8)
Average # of days per treatment episode, Mean (SD)	207.0 (204.5)	108.5 (109.1)	202.9 (189.5)	106.6 (108.5)

Note: This exhibit shows the number of treatment episodes that start with each specific macular degeneration medication. For the primary analysis, treatment episodes are included if they begin with bevacizumab. Since Humana's step therapy policy required bevacizumab as the plan-preferred drug, sub setting to treatment episodes that start with bevacizumab examines the type of medication switching of greatest concern. However, as sensitivity checks, the remaining treatment episodes are examined, whether they start with aflibcerpt, ranibizumab, or other biologic.

© 2024 Liu et al. *JAMA Health Forum*.

eFigure 2. Kaplan-Meier Survivor Curves

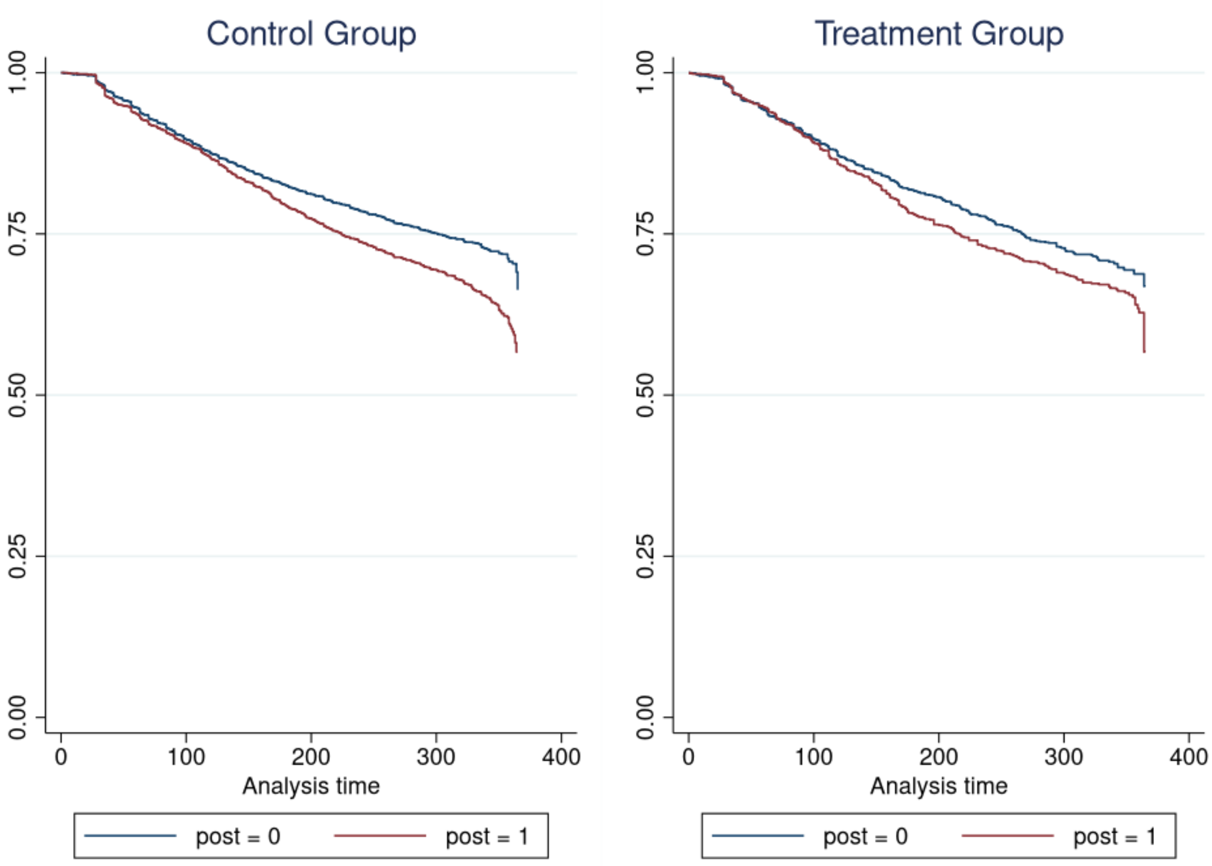

Note: The x-axis is measured in days. The control group are treatment episodes belonging to Aetna and United (left) and the treatment group are treatment episodes belonging to Humana (right). The red lines indicate treatment episodes starting in 2019 and subject to step therapy (for the treatment group), and blue lines indicate treatment episodes starting in 2017 or 2018 and not subject to step therapy. While the treatment episodes belonging to the blue lines could in theory have a look forward period of 1,095 days (or 3 years), the data are subset so that both the red and blue lines have a total look forward period of 365 days (or 1 year). This is to provide consistency between the groups. The Kaplan Meier curves are subset to the sample of treatment episodes that started on bevacizumab during the first administration.

© 2024 Liu et al. *JAMA Health Forum*.

eFigure 3. Parallel Trends Pre-Period Testing for Cox Hazard Regression Model

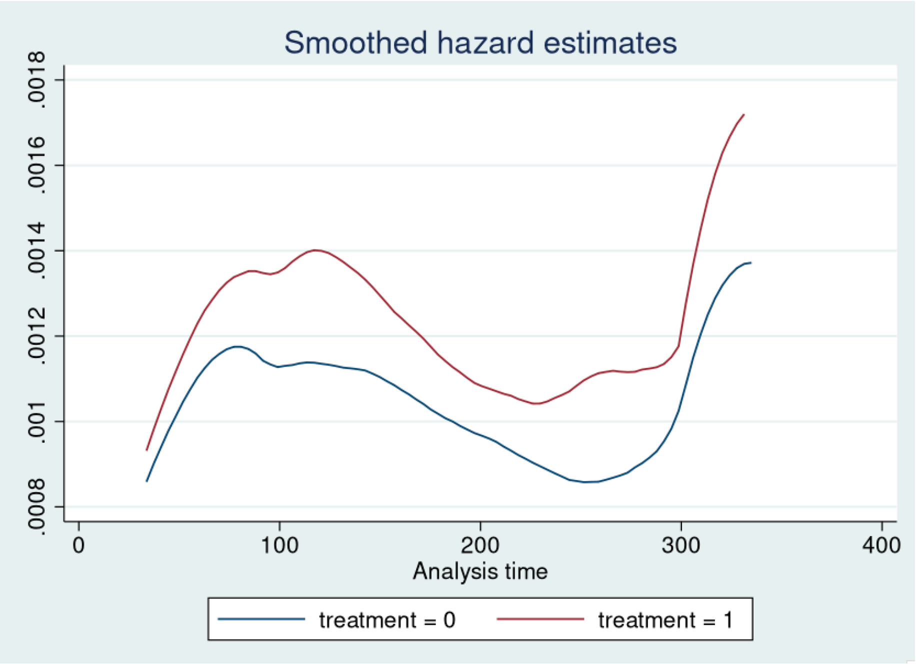

Note**:** To test the parallel trends pre-period assumption within the cox hazard ratio regression framework, we generate hazard functions for the pre period for the control and treatment groups separately. We then graph the smoothed hazard estimates to examine trends in the pre-period. We visually find that the smoothed hazard estimates do not present evidence of non-parallelism in the pre-period.

© 2024 Liu et al. *JAMA Health Forum*.

eFigure 4: Log-log plot to assess proportional hazards assumption

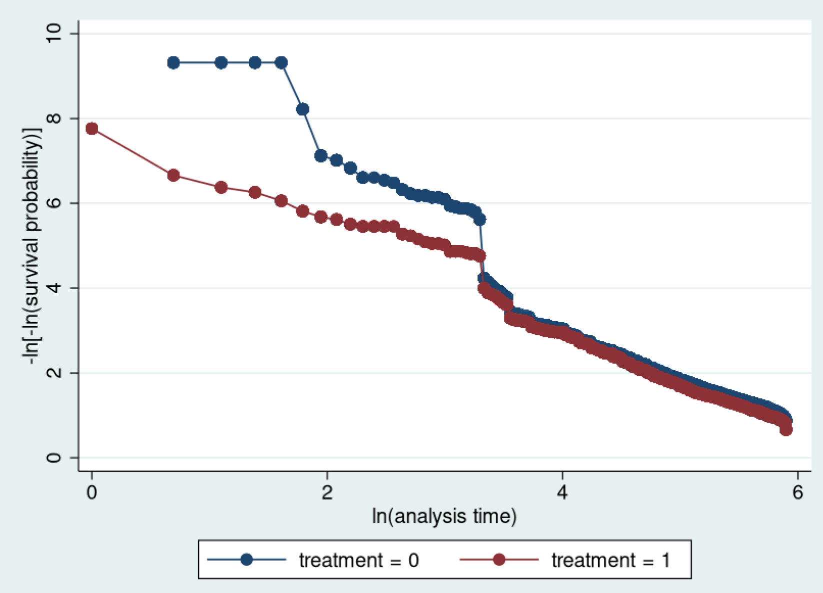

Note: This exhibit depicts visually the log-log curves for the control and treatment groups separately after running the cox hazard ratio regression. We visually see that there is not strong evidence of non-parallelism of the log-log curves.

© 2024 Liu et al. *JAMA Health Forum*.

eTable 7. Cost-savings calculations

Humana 2019 macular degeneration administrations	9,276
Linear Probability Results	0.078
Number of administrations affected due to step therapy (9,276*0.078)	724
Estimated cost savings	
Savings from Aflibercept (362*$908)	+$328,696
Savings from Ranibizumab (362 *$313)	+$113,306
Additional spending from Bevacizumab (724 *64)	-$46,336
**Estimated savings for Humana for 2019**	**+$395,666**
**Scale to 100%**	**+1,978,330**

Note: To estimate cost-savings, the following assumptions are made: 1) the administrations impacted by step therapy, in a counter-factual, would be due to 1,200 administrations changing from aflibercept and ranibizumab administrations to bevacizumab. This may not be the case if the prescribing of bevacizumab changes the demand curve for a macular degeneration administration. Demand for macular degeneration treatment may increase given that bevacizumab is less costly and would result in smaller out of pocket costs for beneficiaries. 2) Of the 1,200 administrations moving away from aflibercept and ranibizumab, they are distributed equally between the two drugs. In practice, aflibercept is prescribed much more prevalently than ranibizumab. Because of the much higher cost of aflibercept, this assumption will result in a conservative estimate. Finally, 3) the price of these drugs is identified through the publicly available Part B dashboard. The negotiated reimbursement for these drugs may by different for MA insurers.

© 2024 Liu et al. *JAMA Health Forum*.
