# Supplementary material for: Macular Degeneration Drug Prescribing Patterns After Step Therapy Introduction in Medicare Advantage
Source: JAMA Health Forum. 2024 Aug 9;5(8):e242446. doi: 10.1001/jamahealthforum.2024.2446 (PMC11316235; doi:10.1001/jamahealthforum.2024.2446)
Supplement: Supplement 2. — Data Sharing Statement [file jamahealthforum-e242446-s002.pdf]

Data Sharing Statement

Liu. Macular Degeneration Drug Prescribing Patterns After Step Therapy Introduction in Medicare Advantage. *JAMA Health Forum*. Published August 09, 2024. doi:10.1001/jamahealthforum.2024.2446

Data
Data available: No

Additional Information
Explanation for why data not available: These data were accessed through a data use agreement with the Center for Medicaid and Medicare Services, and will not be made available.
